# The diagnostic value of GICA used for intraoperative lymph node FNA-Tg measurement to evaluate thyroid cancer metastases

**DOI:** 10.1530/ETJ-23-0182

**Published:** 2024-01-29

**Authors:** Shaodong Hou, Yiceng Sun, Zeyu Yang, Mi Tang, Tingjie Yin, Cong Shao, Cunye Yan, Linlong Mo, Yuquan Yuan, Supeng Yin, Fan Zhang

**Affiliations:** 1 Department of Breast and Thyroid Surgery, Chongqing General Hospital, Chongqing, China; 2 Clinical Medical College, North Sichuan Medical College, Nanchong, Sichuan, China

**Keywords:** papillary thyroid carcinoma, cervical lymph node metastasis, fine needle aspiration, thyroglobulin, colloidal gold-based immunochromatographic assay

## Abstract

**Objective:**

It is crucial to diagnose lymph node (LN) metastases (LNM) before or during thyroid carcinoma surgery. Measurement of thyroglobulin (Tg) in the fine needle aspirate washout (FNA-Tg) is useful to assist in the diagnosis of LNM for papillary thyroid carcinoma (PTC). This study aimed to assess the diagnostic performance of a new technique based on a colloidal gold-based immunochromatographic assay (GICA) for intraoperative FNA-Tg in diagnosing LNM.

**Clinical trial information:**

This study is registered with chictr.org.cn, ID: ChiCTR2200063561 (registered 11 September, 2022).

**Methods:**

This prospective study enrolled 51 PTC patients who underwent cervical LN dissection. A total of 150 LNs dissected from the central and lateral compartments were evaluated by FNA-Tg-GICA at three different time points and compared with frozen sections and the conventional Tg measurement method electrochemiluminescence immunoassay (ECLIA). Receiver operating characteristic curve (ROC) and area under the curve (AUC), cutoff value to discriminate benign and malignant LNs, sensitivity, specificity, and accuracy were provided.

**Results:**

The cutoff value of FNA-Tg to predict LNM was 110.83 ng/mL for ECLIA and 13.19 ng/mL, 38.69 ng/mL, and 77.17 ng/mL for GICA at 3, 10, and 15 min, respectively. There was no significant difference between the AUCs of GICA at different time points compared to using ECLIA and frozen sections. Besides, the diagnostic performance of GICA and ECLIA showed no significant difference in evaluating LNM from central and lateral compartments or between the TgAb-positive subgroup and TgAb-negative subgroup.

**Conclusion:**

GICA is a promising method for intraoperative FNA-Tg measurement and has high value in predicting LNM. It may be a novel alternative or supplementary method to frozen section or ECLIA.

## Introduction

By 2020, thyroid cancer had ranked as the fifth most prevalent cancer in women and the 11th most common cancer overall, with a sharp increase in its frequency worldwide (1). Papillary thyroid carcinoma (PTC) is the most common subtype of thyroid cancer, accounting for 85% of all cases (2). It is estimated that 30–80% of individuals with PTC have cervical lymph node (LN) metastases (LNM) at initial diagnosis (3). Thus, it is crucial to distinguish metastatic LNs from benign LNs before or during surgery, which would help surgeons choose the appropriate surgical scope and prevent over- or undertreatment.

Neck ultrasonography (US) and US-guided fine needle aspiration cytology (FNAC) are routinely used for LNM screening preoperatively. However, the sensitivity and specificity of US in the diagnosis of LNM are limited and rely on the experience of the radiologist to a large extent. It has been reported that preoperative US can only identify malignant central compartment LNs in 20–30% of cases (4, 5). LNs that are suspicious for metastases on US would undergo FNAC. However, the sensitivity of FNAC varies from 75% to 85% with a high rate of nondiagnostic samples (6). The diagnostic efficacy of FNAC may be affected by the tiny size, necrosis, lack of epithelial component in cyst aspirates, or inaccessible position of the LNs (7, 8). During the operation, intraoperative frozen section pathology is usually used for the diagnosis of suspicious LNs. Although the accuracy of frozen sections is relatively high, this approach still has some drawbacks, such as the requirement of some advanced equipment and experienced pathologists.

Thyroglobulin (Tg) is a macromolecular tetrameric glycoprotein with a molecular weight of 600 kDa and is produced and released by thyroid follicular epithelial cells (9). In 1992, Pacini *et al.* first suggested that elevated concentrations of Tg in fine needle aspirate washout (FNA-Tg) of nonthyroidal neck nodes could assist in the diagnosis of metastases from differentiated thyroid cancer (DTC) (6). Since then, a variety of studies have demonstrated the high sensitivity (92–95%) and specificity (91–93%) of this method in the diagnosis of LNM (3, 8, 10, 11, 12, 13).

Electrochemiluminescence immunoassay (ECLIA) is the most common approach used for Tg measurement because of its high reproducibility, sensitivity, and stability (14). However, this assay for Tg testing requires expensive equipment and specially trained technical staff, is generally carried out in the laboratory, and takes several hours. Currently, a new technique based on colloidal gold-based immunochromatographic assay (GICA) for Tg measurement has been developed. The device used by GICA is portable and can be placed in the operating room. This novel method for Tg measurement is quantitative, easy to operate, and time-saving, as it works within a few minutes.

In this study, we used GICA to measure FNA-Tg intraoperatively and evaluated its efficacy in diagnosing LNM of PTC by comparison with ECLIA and frozen section. We also explored the cutoff value of FNA-Tg and the optimum timing of GICA to diagnose LNM. It may provide a novel, accurate, convenient, and rapid method for LNM diagnosis during thyroidectomy.

## Materials and methods

### Patients

This study enrolled 51 PTC patients who underwent therapeutic or prophylactic cervical LN dissection between September 2022 and January 2023 in the Department of Breast and Thyroid Surgery of Chongqing General Hospital. The LNs were divided into two groups: the central compartment group and the lateral compartment group. The study was approved by the ethics committee of Chongqing General Hospital (Chongqing, China) and was registered with the Chinese Clinical Trial Register (www.chictr.org.cn, identifier ChiCTR2200063561). Written informed consent was obtained from all individual participants included in the study.

### FNA procedure

**Figure 1:**
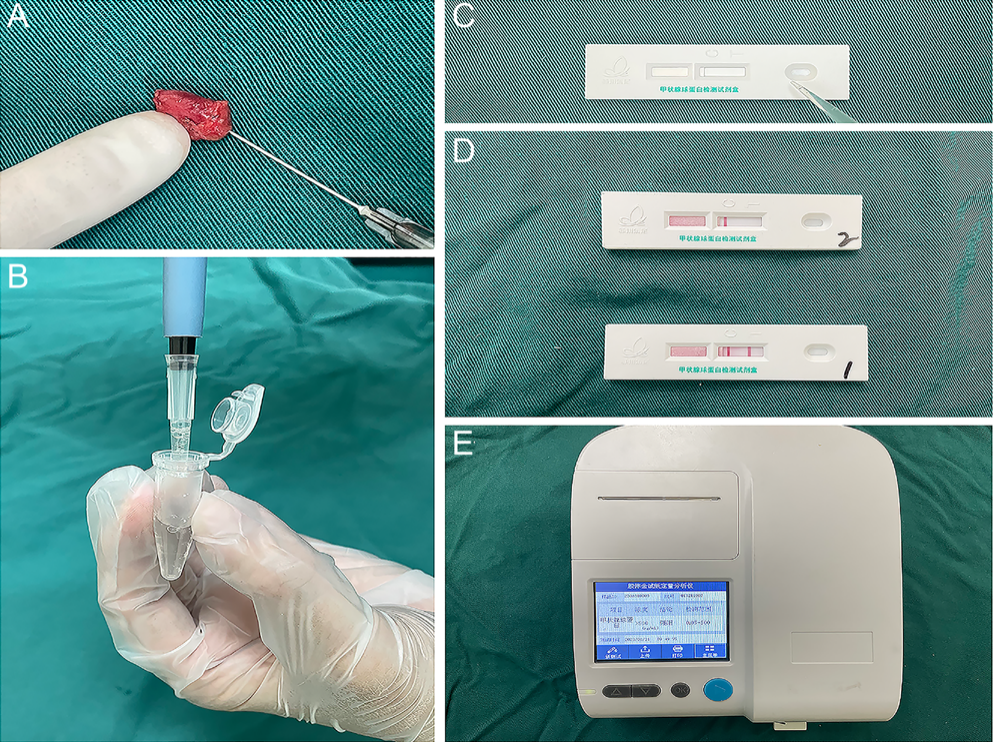
The detection procedure for GICA assay. A. A lymph node is aspirated along its long axis using a thin 23G needle. B andC. The washout sample is then deposited into the hole of a test strip. Through capillary action, the Tg in the sample flows along the strip and reacts with anti-Tg antibodies embedded on the strip’s membrane. D. The reaction in the test zone on the membrane. If Tg is present in the sample, the test zone turns red (bottom strip), while a negative sample leaves it blank (upper strip). E. The intensity of the color is measured by an instrument to quantify the concentration of Tg.

During the operation, after the LNs of the central compartment or lateral compartment were dissected, an experienced surgeon selected one or two macroscopically abnormal and one or two apparently normal LNs in every patient. FNA was performed using a 23G needle with a 5 mL syringe. The needle was repeatedly moved four to six times inside each LN along its major axis until the needle hub was filled with LN tissue. Immediately after aspiration, the needle and syringe were washed with 1 mL of saline solution. Then, 80 μL of washout fluid were pipetted for Tg measurement by GICA (Fig. 1A and B). The remaining washout was sent to the laboratory for Tg measurement by ECLIA using a commercially available kit (Roche E170 Modular Immunoassay Analyzer). The Tg-ECLIA method was registered by the vendor exclusively for serum samples. Based on previous studies (15, 16, 17), our clinical practice data consistently confirm that ECLIA is an effective tool for the detection of FNA-Tg, the assay’s quantification range for Tg concentration spans from 0 to 481 ng/mL.

### GICA for Tg measurement

The Tg-GICA rapid test kit was developed by Suzhou Dongni Biotechnology Co., Ltd (Suzhou, Jiangsu Province, China). The principle of GICA is to use colloidal gold as a tracer marker in antigen–antibody reactions. The Tg detection kit consists of plastic boxes and test strips installed in the box. Each test strip mainly consists of a sample pad, a conjugate pad, an absorbent pad, a test line, and a control line on a nitrocellulose membrane. An existing gold-labeled antibody on the nitrocellulose membrane is the pre-coating of mouse anti-human Tg monoclonal antibody within the test zone (T). Pre-embedded in the control zone (C) is the goat anti-mouse IgG polyclonal antibody. At the time of testing, 80 μL of FNA washout fluid were added to the sample pad and was allowed to flow toward the absorption pad through the capillary effect. Tg in the sample first binds to the colloidal gold–antibody conjugates that are precoated on the conjugate pad, and then the complexes are captured by the Tg antibody located in the test line and form a pink or red ribbon, and the degree of coloration of the ribbon is proportional to the Tg level in the sample tested within a certain range. The control line is precoated with anti-streptavidin antibody and should always become red. Our pre-experiments and previous studies of qualitative protein detection using GICA methods can yield stable results between 3 and 15 min (18, 19). We thus selected 3, 10, and 15 min as three time points for our experiments. The test strips were quantitatively analyzed using an instrument (Ma'anshan Ruiheng Technology Co., China) at each of the selected time points following sample addition. This instrument comprises a motherboard, a connecting plate, a serial port, an optical system, a touch screen, and a software suite. The image acquisition module captures an image of the test area and converts the image into a photoelectric signal by recognizing the difference in depth between the C and T zones on the gold-labeled strip to garner sample data. The concentration of Tg measured by this instrument ranges from 0.05 to 500 ng/mL (Fig. 1C, D, and E).

### Histopathological diagnosis

Each LN that underwent FNA was separately sent for intraoperative frozen sectioning and finally diagnosed by paraffin sectioning, which is the gold standard for diagnosing LNM.

### Statistical analysis

The value of FNA-Tg determined by ECLIA and GICA at different time points, the preoperative level of serum TgAb, and the pathology of frozen and paraffin sections of each patient were recorded. The data were analyzed and plotted using MedCalc program version 15.2 (MedCalc Software, Ostend, Belgium) and R software (v4.2.1). Continuous data were recorded as the mean and standard deviation. Categorical data were recorded as counts and percentages. The ROC curve and the AUC of the two tests are displayed. McNemar’s test was used to determine the sensitivity and specificity. The DeLong test was used to compare diagnostic performance, and the Jorden index was calculated to find the best diagnostic cutoff value for the diagnosis of LNM by FNA-Tg. The consistency between the GICA-Tg and ECLIA-Tg methods was examined by linear regression analysis. In all analyses, a two-sided* P* value < 0.05 was considered to be statistically significant.

## Results

### Patient characteristics

Fifty-one PTC patients were included in this study with a mean age of 39.15 years (±10.96 years), of whom 34 (66.67%) were female. In total, 10 patients had undergone reoperation, while 41 were receiving their first thyroid surgery. The mean body mass index of all the patients was 23.92 (± 3.36). According to the 8th American Joint Committee on Cancer (AJCC) tumor, node, and metastases (TNM) system, there were 35 pT1, 14 pT2, and 2 pT3 patients. Forty-five patients were classified as stage I, and six patients were classified as stage II. None had received prior radioactive iodine therapy.

A total of 150 LNs from the 51 patients were analyzed, including 72 (48.00%) metastatic LNs and 78 (52.00%) benign LNs that were diagnosed by paraffin section, with mean sizes of 0.88 cm (±0.38 cm) and 0.63 cm (±0.31 cm), respectively. Among the 150 LNs, 59 were dissected from the central compartment, and 91 were dissected from the lateral compartment (The basic information of patients and LNs is detailed in Supplementary Table 1, see the section on supplementary materials given at the end of this article.).

### Diagnostic performance of intraoperative FNA-Tg-GICA and FNA-Tg-ECLIA for the total group LNs

**Figure 2:**
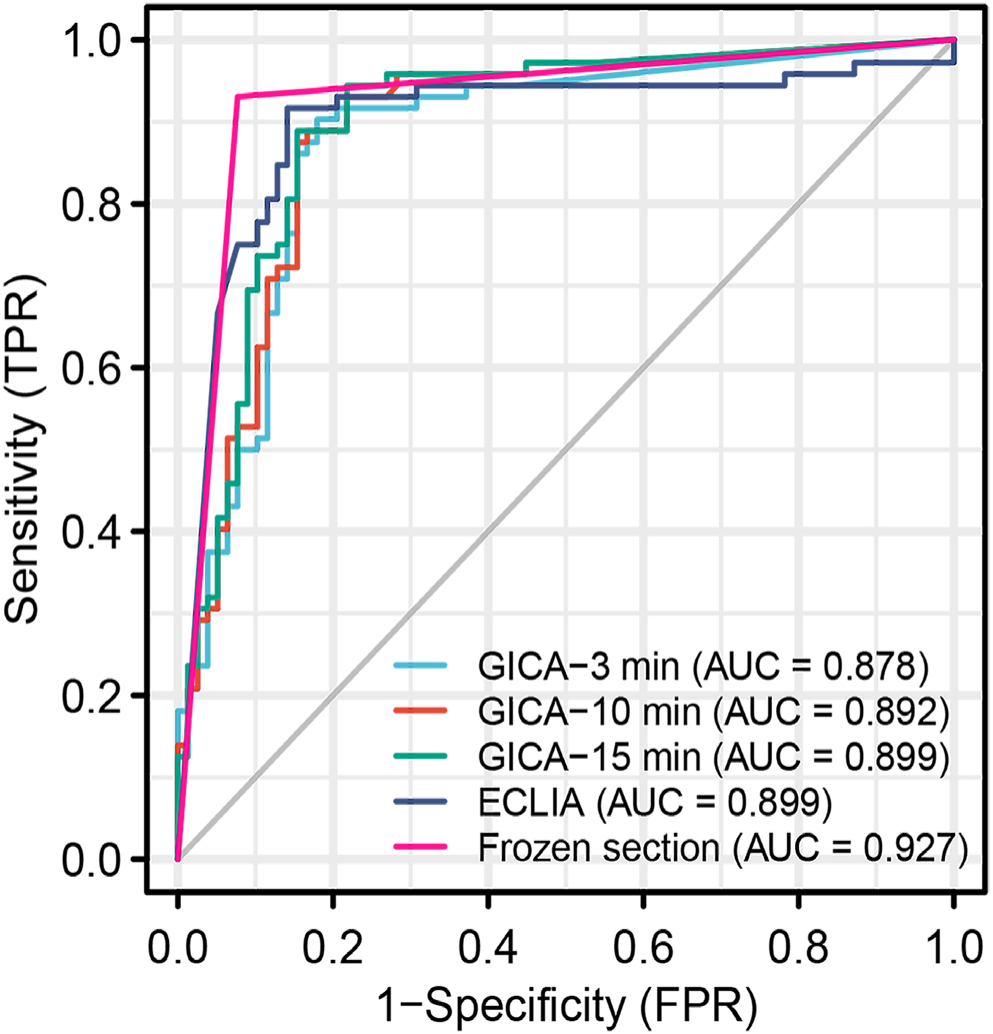
ROC curves of GICA at different time points, ECLIA, and frozen section for the total group lymph nodes. The DeLong test showed no significant differences between GICA at different time points and ECLIA (*P* = 0.196), between GICA-3 min and frozen section (*P* = 0.051), and between ECLIA and frozen section (*P* = 0.198).

**Figure 3:**
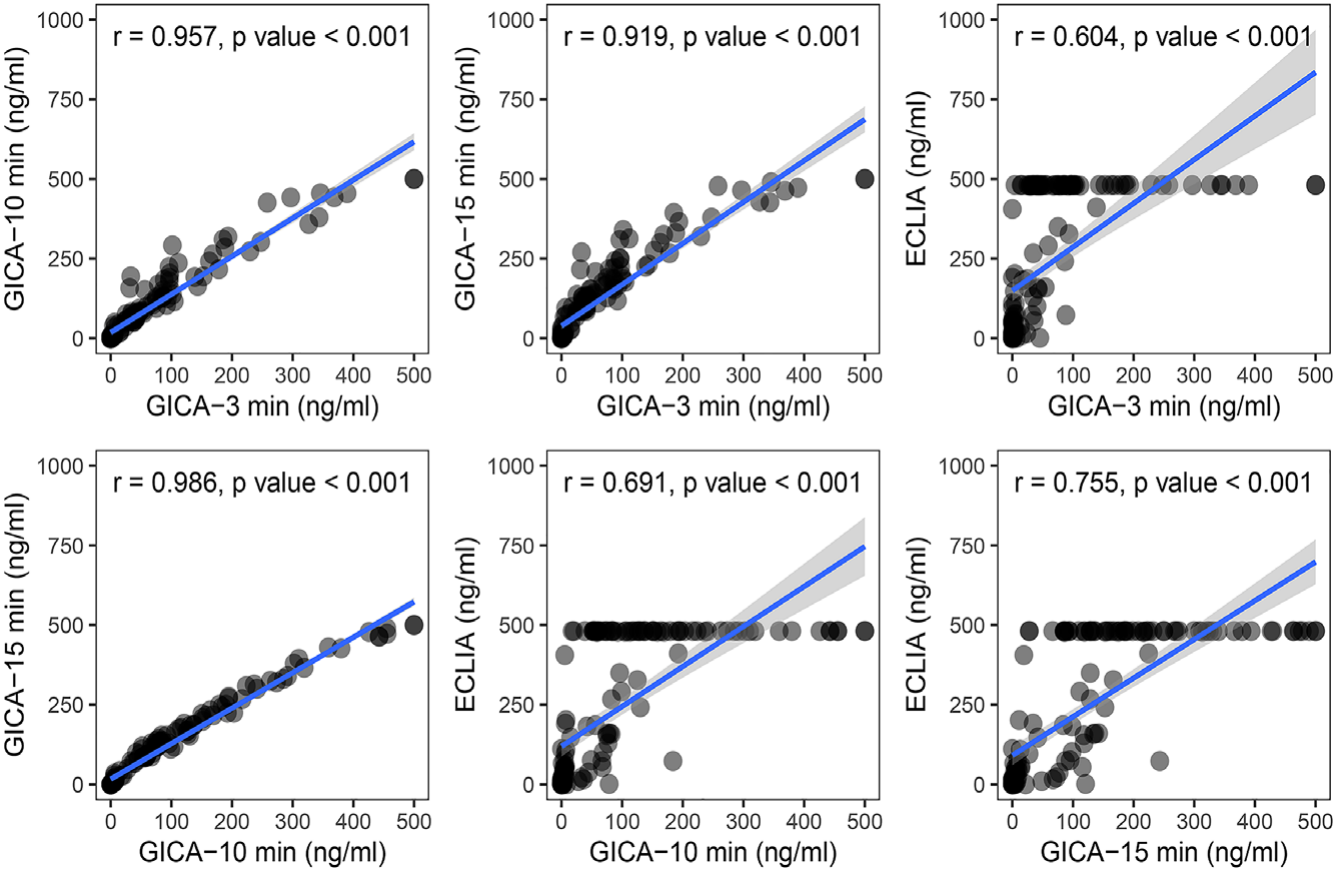
Consistency between GICA at different time points and ECLIA. The Spearman’s correlation analysis showed a linear relationship between the Tg measurement results detected by GICA and ECLIA. The upper reading of the ECLIA was 481 ng/mL.

**Figure 4:**
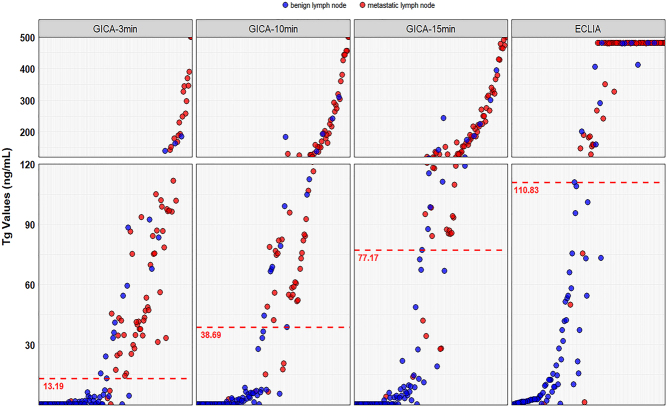
Tg values of each LN measured by GICA at different time points and ECLIA. Pathologically proven metastatic lymph nodes are denoted by red dots, and benign lymph nodes are represented by blue dots. The dotted line indicates the diagnostic cutoff value for each method.

When using GICA, according to the level of FNA-Tg measured after adding samples at 3, 10, and 15 min, the AUCs of the ROC curves of this method to identify LNM were 0.878 (95% CI: 0.815–0.926), 0.892 (95% CI: 0.831–0.936), and 0.899 (95% CI: 0.839–0.942), respectively. The AUC of the ROC curve of ECLIA to identify LNM was 0.899 (95% CI: 0.839–0.942) (Table 1). The AUCs of GICA at different time points and ECLIA were compared by the DeLong test, and the results showed that there was no significant difference between these groups (*P* = 0.196) (Fig. 2). To validate the GICA-Tg assay, Spearman correlation analysis was conducted between GICA-Tg and ECLIA-Tg measurements at 3, 10, and 15 min. Spearman’s correlation analysis showed the relationship between the Tg measurement results detected by GICA at different time points and ECLIA methods, revealed a robust positive correlation between the two methods (Fig. 3). These results indicate that both GICA and ECLIA can efficiently diagnose LNM intraoperatively, with 3 min being enough to achieve an ideal diagnostic performance for GICA. Therefore, we mainly used the data of GICA-3 min for the next analyses. In addition, according to the ROC curves (Fig. 2), the optimal cutoff value of FNA-Tg to predict LNM was 110.83 ng/mL for ECLIA and 13.19 ng/mL, 38.69 ng/mL, and 77.17 ng/mL for GICA at 3, 10, and 15 min, respectively (Table 1). Scatter plots were generated by comparing GICA-Tg and ECLIA-Tg measurements against each LN proven by paraffin, with the diagnostic cutoff marked by dotted lines (Fig. 4).

**Table 1:** Comparison of diagnostic performance of intraoperative FNA-Tg-GICA and FNA-Tg-ECLIA for the total group LNs.

	Cutoff value (ng/mL)	Sensitivity^a^	Specificity^a^	Accuracy^a^	AUC^a^	*P*
GICA						0.196^b^
3 min	13.19	0.90 (0.81–0.96)	0.82 (0.72–0.90)	0.85 (0.79–0.90)	0.878 (0.815–0.926)	
10 min	38.69	0.89 (0.79–0.95)	0.83 (0.73–0.91)	0.86 (0.79–0.90)	0.892 (0.831–0.936)	
15 min	77.17	0.89 (0.79–0.95)	0.85 (0.75–0.92)	0.86 (0.80–0.91)	0.899 (0.839–0.942)	
ECLIA	110.83	0.92 (0.83–0.97)	0.86 (0.76–0.93)	0.88 (0.82–0.93)	0.899 (0.839–0.942)	

^a^Values in parentheses are 95% CI; ^b^*P* value for the comparison of AUCs between GICA at different time points and ECLIA.

AUC, area under the curve; ECLIA, electrochemiluminescence immunoassay; GICA, colloidal gold-based immunochromatographic assay.

### Diagnostic performance of FNA-Tg-GICA, FNA-Tg-ECLIA, and intraoperative frozen section

All LNs subjected to FNA were sent for intraoperative frozen sectioning and finally analyzed by paraffin sectioning. The final histopathological diagnosis of the LNs showed that there were 72 metastatic LNs and 78 benign LNs, and the frozen section results showed 73 metastatic LNs and 77 benign LNs. The sensitivity, specificity, and accuracy of frozen sections were 0.93 (95% CI: 0.85–0.98), 0.92 (95% CI: 0.84–0.97), and 0.92 (95% CI: 0.87–0.96), respectively. The diagnostic performance of FNA-Tg-GICA, FNA-Tg-ECLIA, and intraoperative frozen section was compared by DeLong’s test. The results showed that there was no significant difference among the three methods to diagnose LNM intraoperatively (Table 2 and Fig. 2), suggesting that FNA-Tg-GICA and FNA-Tg-ECLIA are as accurate as frozen sections in diagnosing LNM.

**Table 2:** Comparison of diagnostic performance of FNA-Tg.-GICA, FNA-Tg-ECLIA, and intraoperative frozen section. Data are presented as value (95% CI).

	Sensitivity	Specificity	Accuracy	AUC	*P*	
GICA-3 min	0.90 (0.81–0.96)	0.82 (0.72–0.90)	0.85 (0.79–0.90)	0.878 (0.815–0.926)		
ECLIA	0.92 (0.83–0.97)	0.86 (0.76–0.93)	0.88 (0.82–0.93)	0.899 (0.839–0.942)		
Frozen section	0.93 (0.85–0.98)	0.92 (0.84–0.97)	0.92 (0.87–0.96)	0.927 (0.873–0.963)	0.051^a^	0.198^b^

^a^Value for the comparison of AUCs between GICA-3 min and frozen section. ^b^Value for the comparison of AUCs between ECLIA and frozen section.;

AUC, area under the curve; ECLIA, electrochemiluminescence immunoassay; GICA, colloidal gold-based immunochromatographic assay.

### Diagnostic performance of intraoperative FNA-Tg-GICA and FNA-Tg-ECLIA for the central and lateral compartment LNs

In the treatment of thyroid carcinoma, surgeons decide the surgical scope of LN dissection based on pathologically diagnosed or suspicious LNM in the different regions, which mainly include the central compartment and lateral compartment. Therefore, we explored whether the diagnostic performance of GICA and ECLIA is different for LNs from these two regions. Overall, the diagnostic performance of both Tg measurement methods showed no significant difference in evaluating LNM in the central and lateral compartment group (Table 3), implying that FNA-Tg-GICA and FNA-Tg-ECLIA are efficient in diagnosing LNM in both the central and lateral compartment.

**Table 3:** Diagnostic performance of FNA-Tg-GICA and FNA-Tg-ECLIA for the central compartment and lateral compartment LNs. Data are presented as value (95% CI).

	Sensitivity	Specificity	Accuracy	AUC	*P*
GICA-3 min					0.559^a^
Central compartment	0.88 (0.62–0.98)	0.79 (0.63–0.89)	0.81 (0.69–0.89)	0.834 (0.714–0.918)	
Lateral compartment	0.91 (0.79–0.97)	0.83 (0.67–0.93)	0.88 (0.79–0.93)	0.871 (0.785–0.932)	
ECLIA					0.968^b^
Central compartment	0.94 (0.69–1.00)	0.83 (0.68–0.92)	0.86 (0.75–0.93)	0.887 (0.778–0.955)	
Lateral compartment	0.91 (0.79–0.97)	0.86 (0.70–0.95)	0.89 (0.81–0.94)	0.885 (0.801–0.942)	

^a^*P* value for the comparison of AUCs of GICA-3 min between LNs from central compartment and lateral compartment. ^b^*P* value for the comparison of AUCs of ECLIA between LNs from central compartment and lateral compartment.

AUC, area under the curve; ECLIA, electrochemiluminescence immunoassay; GICA, colloidal gold-based immunochromatographic assay.

### Serum TgAb levels and the diagnostic performance of FNA-Tg

Of the 51 patients, 18 were TgAb positive, and 33 were TgAb negative. Fifty-two LNs were from TgAb-positive patients, and 98 LNs were from TgAb-negative patients. In the TgAb-positive subgroup, a diagnosis based on GICA had a sensitivity of 0.88 (95% CI: 0.69–0.97), a specificity of 0.81 (95% CI: 0.60–0.93), and an accuracy of 0.85 (95% CI: 0.72–0.92). The corresponding values in the TgAb-negative subgroup were 0.91 (95% CI: 0.78–0.97), 0.81 (95% CI: 0.67–0.90), and 0.86 (95% CI: 0.77–0.91), respectively. For ECLIA, the sensitivity, specificity, and accuracy were 0.88 (95% CI: 0.69–0.97), 0.88 (95% CI: 0.69–0.97), and 0.88 (95% CI: 0.77–0.95) in the TgAb-positive subgroup and 0.93 (95% CI: 0.81–0.98), 0.83 (95% CI: 0.69–0.91), and 0.88 (95% CI: 0.80–0.93) in the TgAb-negative subgroup, respectively (Table 4). The AUCs of both methods showed no significant difference between the TgAb-positive subgroup and the TgAb-negative subgroup (Table 4), suggesting that the presence of serum TgAb does not affect the diagnostic performance of GICA and ECLIA.

**Table 4:** Diagnostic performance of intraoperative FNA-Tg in the presence and absence of TgAb. Data are presented as value (95% CI).

	Sensitivity	Specificity	Accuracy	AUC	*P*
GICA-3 min					0.818^a^
TgAb-negative	0.91 (0.78–0.97)	0.81 (0.67–0.90)	0.86 (0.77–0.91)	0.860 (0.776–0.922)	
TgAb-positive	0.88 (0.69–0.97)	0.81 (0.60–0.93)	0.85 (0.72–0.92)	0.846 (0.719–0.931)	
ECLIA					0.946^b^
TgAb-negative	0.93 (0.81–0.98)	0.83 (0.69–0.91)	0.88 (0.80–0.93)	0.881 (0.800–0.938)	
TgAb-positive	0.88 (0.69–0.97)	0.88 (0.69–0.97)	0.88 (0.77–0.95)	0.885 (0.766–0.956)	

^a^*P* value for the comparison of AUCs of GICA-3 min between LNs from TgAb-negative and TgAb-positive patients. ^b^*P* value for the comparison of AUCs of ECLIA between LNs from TgAb-negative and TgAb-positive patients.

AUC, area under the curve; ECLIA, electrochemiluminescence immunoassay; GICA, colloidal gold-based immunochromatographic assay.

## Discussion

Cervical LNM is an independent risk factor for local recurrence of thyroid carcinoma (20). For patients with PTC, the accurate assessment of LNM preoperatively or intraoperatively is vital to determine the appropriate surgical scope and help the surgeon decide whether to perform a therapeutic cervical LN dissection. Recently, FNA-Tg has been shown to be an efficient technique that can improve the diagnostic accuracy of US and FNAC in evaluating LNM in PTC. It is recommended in both the American Thyroid Association and European Thyroid Association thyroid cancer treatment guidelines (3, 13). However, most of the previous studies performed FNA-Tg preoperatively or postoperatively. Only a few studies have assessed its diagnostic value for LNM intraoperatively (21).

In this study, we have introduced a novel method based on GICA to measure FNA-Tg and assist in the diagnosis of LNM intraoperatively. The results demonstrated that GICA is a rapid, easy-to-operate, and accurate method to diagnose TNM compared with the conventional approach of ECLIA and frozen sections. Although without significant difference, the *P* value for the comparison of AUCs between GICA-3 min and frozen section was close to 0.05, and the specificity of frozen section also seems higher, which indicates that the diagnostic performance of FNA-Tg tested by GICA may be slightly inferior to the frozen section. However, frozen sections require specialized equipment and experienced pathologists and often take a relatively longer time. The advantages of GICA may make it a novel alternative or supplementary method for LNM diagnosis intraoperatively, especially for hospitals that are unable to perform ECLIA or frozen section.

Some meta-analyses showed that the pooled sensitivity and specificity of FNA-Tg preoperatively or postoperatively were approximately 93–95% and 92–94.5%, respectively (8, 12). However, in this study, the diagnostic performance of FNA-Tg-GICA and FNA-Tg-ECLIA intraoperatively seemed poorer than that of FNA-Tg preoperatively or postoperatively, especially for specificity. This discrepancy may be attributed to the following reasons. On the one hand, all of the LNs in this study were subjected to paraffin sectioning to obtain the final histological diagnosis, but many previous reports diagnosed benign LNs only by FNAC without histology (10, 22, 23, 24, 25). As FNAC is less accurate than paraffin section, this may misclassify some false-negative cases as true-negative cases. On the other hand, previous studies focused more on the lateral compartment LNs (26, 27). However, in this study, central compartment LNs accounted for approximately 40% of the total, and these LNs were more likely to be contaminated by thyroid tissue during a puncture, leading to more false-positive cases.

It is widely recognized that TgAb negatively affects the immunoassay measurement of serum Tg, but it is still controversial whether TgAb affects the results of FNA-Tg. Baskin first proposed that FNA-Tg was not influenced by positive serum TgAb, possibly because intracellular Tg is not exposed to circulating TgAb (28). Marta *et al.* reported that the values of FNA-Tg in patients with LNM did not differ between those with positive and those with negative TgAb (29). In contrast, Jeon *et al.* discovered that FNA-Tg levels in the LNs of serum TgAb-positive patients were significantly lower than those of TgAb-negative patients, and the sensitivity and negative predictive value of FNA-Tg diagnosis were lower in the TgAb-positive group than in the TgAb-negative group (30). In agreement with most previous studies, our results demonstrated that the presence of serum TgAb does not affect the diagnostic performance of GICA and ECLIA, suggesting that GICA is as stable as other immunoassays for Tg measurement.

There are some limitations of our study. First, a selection bias may have existed, and the number of LNs from the lateral compartment was much greater than that from the central compartment. In addition, most of the values of Tg detected by GICA were much lower than those detected by ECLIA for the same LN. This implies that GICA may not be so precise in Tg quantification analysis but can still be used as a semiquantitative or qualitative method to evaluate LNM intraoperatively. When the Tg levels in washout fluid exceeded the highest limit of 481 ng/mL measured by ECLIA, we did not dilute the samples. This led to limited correlation between GICA and ECLIA.

In conclusion, our results support that GICA is a promising method for intraoperative FNA-Tg measurement to predict LNM. It may be a novel alternative or supplementary method to frozen section or ECLIA for LNM diagnosis intraoperatively.

## Supplementary materials

Supplementary Table 1

## Declaration of interest

The authors declare that there is no conflict of interest that could be perceived as prejudicing the impartiality of the study reported.

## Funding

This work was supported by the Chongqing Medical Scientific Research Project (Joint Project of Chongqing Health Commission and Science and Technology Bureau) (Grant no. 2023QNXM017) and Key Special Project for Technological Innovation and Application Development of Chongqing (Grant no. CSTB2022TIAD-KPX0177).

## Author contribution statement

The authors have made the following declarations about their contributions: FZ and YS conceived and designed this study; SY, ZY, MT, TY, CS, CY, LM, and YY performed the trial and collected the data; SY, YS, and SH analyzed the data; and FZ, SH, and YS drafted the manuscript.
