# Supplementary material for: The diagnostic value of GICA used for intraoperative lymph node FNA-Tg measurement to evaluate thyroid cancer metastases
Source: Eur Thyroid J. 2024 Jan 29;13(1):e230182. doi: 10.1530/ETJ-23-0182 (PMC10895302; doi:10.1530/ETJ-23-0182)
Supplement: Supplementary Table 1 [file supplementary_table_1.pdf]

| Patient number | Reoperation | Gender | Year | BMI | Tumor size (cm) | TgAb (IU/ml) | Lymph Node number | Longest (cm) | Location | GICA-3 min (ng/ml) | GICA-10 min (ng/ml) | GICA-15 min (ng/ml) | ECLIA (ng/ml) | Frozen section | Pathology |
|---|---|---|---|---|---|---|---|---|---|---|---|---|---|---|---|
| Patient1 | Yes | Male | 36 | 25 | 2 | 12 | LN1 | 1.0 | Lateral | 86.36 | 129.94 | 152.63 | 241.53 | Metastatic | Metastatic |
| | | | | | | | LN2 | 0.6 | Lateral | 75.23 | 95.86 | 128.37 | 349.5 | Metastatic | Metastatic |
| Patient2 | No | Female | 22 | 18.1 | 4.5 | 0.1 | LN3 | 0.8 | Lateral | 389.64 | 455.57 | 472.73 | 481 | Metastatic | Metastatic |
| | | | | | | | LN4 | 0.2 | Lateral | 296.73 | 442.29 | 464.28 | 481 | Metastatic | Metastatic |
| Patient3 | No | Female | 30 | 29.1 | 4 | 0.18 | LN5 | 0.8 | Lateral | 193.42 | 318.88 | 365.71 | 481 | Metastatic | Metastatic |
| | | | | | | | LN6 | 0.6 | Lateral | 4.33 | 38.69 | 66.62 | 481 | Benign | Benign |
| Patient4 | No | Female | 41 | 22.9 | 1.2 | 3.53 | LN7 | 1.0 | Central | 0.05 | 0.05 | 0.05 | 3.34 | Benign | Benign |
| | | | | | | | LN8 | 0.5 | Lateral | 0.05 | 0.05 | 0.05 | 2.42 | Benign | Benign |
| Patient5 | Yes | Female | 35 | 24.8 | 2.6 | 0.1 | LN9 | 1.5 | Central | 1.66 | 2.5 | 3.41 | 3.79 | Benign | Benign |
| | | | | | | | LN10 | 1.0 | Lateral | 500 | 500 | 500 | 481 | Metastatic | Metastatic |
| | | | | | | | LN11 | 1.6 | Lateral | 168.42 | 263.11 | 324.75 | 481 | Metastatic | Metastatic |
| Patient6 | Yes | Female | 32 | 25.4 | 1.6 | 2.42 | LN12 | 1.3 | Lateral | 93.42 | 125.05 | 166.38 | 327.2 | Metastatic | Metastatic |
| | | | | | | | LN13 | 1.0 | Central | 17.34 | 42.15 | 98.33 | 181.6 | Metastatic | Metastatic |
| | | | | | | | LN14 | 0.3 | Lateral | 0.05 | 0.05 | 0.05 | 2.74 | Benign | Benign |
| Patient7 | Yes | Female | 27 | 22.3 | 1.4 | 4.67 | LN15 | 0.8 | Lateral | 188.27 | 284.66 | 330.64 | 481 | Metastatic | Metastatic |
| | | | | | | | LN16 | 1.3 | Lateral | 343.6 | 379.73 | 426.63 | 481 | Metastatic | Metastatic |
| | | | | | | | LN17 | 1.5 | Lateral | 1.32 | 2.18 | 3.77 | 6.31 | Benign | Benign |
| Patient8 | Yes | Female | 26 | 20.2 | 4.6 | 5.66 | LN18 | 0.6 | Lateral | 104.83 | 116.26 | 177.04 | 481 | Metastatic | Metastatic |
| | | | | | | | LN19 | 0.3 | Lateral | 88.29 | 183.73 | 243.03 | 73.1 | Benign | Benign |
| | | | | | | | LN20 | 0.5 | Lateral | 0.05 | 0.05 | 0.05 | 9.74 | Benign | Benign |
| | | | | | | | LN21 | 0.5 | Lateral | 0.05 | 0.05 | 0.05 | 0.12 | Benign | Benign |
| Patient9 | No | Female | 24 | 23.3 | 3.5 | 0.7 | LN22 | 0.5 | Lateral | 500 | 500 | 500 | 481 | Metastatic | Metastatic |

| | | | | | | | | | | | | | | | |
|---|---|---|---|---|---|---|---|---|---|---|---|---|---|---|---|
| | | | | | | | LN23 | 1.3 | Lateral | 69.78 | 106.56 | 163.1 | 481 | Metastatic | Metastatic |
| | | | | | | | LN24 | 0.4 | Lateral | 258.04 | 425.94 | 478.12 | 481 | Metastatic | Metastatic |
| | | | | | | | LN25 | 1.3 | Lateral | 143.21 | 164.17 | 231.81 | 481 | Metastatic | Metastatic |
| Patient10 | Yes | Female | 30 | 19.5 | 3.8 | 0.04 | LN26 | 0.7 | Central | 48.62 | 84.04 | 153.26 | 481 | Metastatic | Metastatic |
| | | | | | | | LN27 | 0.5 | Lateral | 101.68 | 291.63 | 339.8 | 481 | Metastatic | Metastatic |
| | | | | | | | LN28 | 0.6 | Lateral | 45.32 | 78.58 | 120.69 | 1.27 | Benign | Metastatic |
| Patient11 | Yes | Male | 40 | 24.8 | 1.2 | 90.52 | LN29 | 1.1 | Central | 43.08 | 74.74 | 117.94 | 154.14 | Metastatic | Metastatic |
| | | | | | | | LN30 | 1.3 | Central | 34.58 | 82.41 | 128.36 | 266.95 | Metastatic | Metastatic |
| | | | | | | | LN31 | 0.6 | Central | 1.12 | 1.46 | 2.19 | 0.43 | Benign | Benign |
| Patient12 | No | Female | 29 | 18.4 | 2.0 | 115.53 | LN32 | 0.5 | Lateral | 3.13 | 15.06 | 41.92 | 146.91 | Metastatic | Metastatic |
| | | | | | | | LN33 | 1.1 | Lateral | 84.2 | 126.8 | 157.79 | 481 | Metastatic | Metastatic |
| | | | | | | | LN34 | 0.6 | Lateral | 0.05 | 0.05 | 0.05 | 9.28 | Benign | Benign |
| Patient13 | No | Female | 56 | 24.9 | 3.0 | 0.02 | LN35 | 0.4 | Lateral | 0.05 | 0.05 | 0.05 | 2.46 | Benign | Benign |
| | | | | | | | LN36 | 0.9 | Lateral | 0.05 | 0.05 | 0.05 | 1 | Benign | Benign |
| | | | | | | | LN37 | 1.3 | Lateral | 46.75 | 81.93 | 134.23 | 481 | Metastatic | Metastatic |
| Patient14 | No | Male | 42 | 29.8 | 1.1 | 0.15 | LN38 | 0.7 | Lateral | 35.86 | 67.41 | 115.32 | 54.19 | Benign | Benign |
| | | | | | | | LN39 | 1.0 | Lateral | 101.94 | 136.92 | 184.91 | 481 | Metastatic | Metastatic |
| | | | | | | | LN40 | 0.8 | Lateral | 0.05 | 0.05 | 0.05 | 1.09 | Benign | Benign |
| Patient15 | No | Female | 52 | 21.9 | 2.0 | 298.03 | LN41 | 0.6 | Lateral | 0.05 | 0.05 | 0.05 | 1.35 | Benign | Benign |
| | | | | | | | LN42 | 0.6 | Lateral | 34.34 | 76.74 | 118.15 | 128.85 | Metastatic | Metastatic |
| | | | | | | | LN43 | 0.4 | Lateral | 0.05 | 0.05 | 0.05 | 1.49 | Benign | Benign |
| Patient16 | No | Male | 48 | 26 | 1.7 | 0.03 | LN44 | 0.5 | Lateral | 345.62 | 455.06 | 489.92 | 481 | Metastatic | Metastatic |
| | | | | | | | LN45 | 0.5 | Lateral | 0.05 | 0.05 | 0.05 | 4.37 | Benign | Benign |
| Patient17 | No | Female | 27 | 19.6 | 2.5 | 0.06 | LN46 | 0.8 | Lateral | 247.3 | 302.4 | 378.71 | 481 | Metastatic | Metastatic |
| | | | | | | | LN47 | 1.3 | Lateral | 53.18 | 84.94 | 136.21 | 481 | Metastatic | Metastatic |

| | | | | | | | | | | | | | | | |
|---|---|---|---|---|---|---|---|---|---|---|---|---|---|---|---|
| | | | | | | | LN48 | 0.3 | Lateral | 96.62 | 179.07 | 249.64 | 481 | Metastatic | Metastatic |
| | | | | | | | LN49 | 0.7 | Lateral | 98.75 | 151.25 | 198.53 | 481 | Metastatic | Metastatic |
| Patient18 | No | Female | 57 | 24.8 | 0.5 | 0.02 | LN50 | 1.0 | Lateral | 7.18 | 33.26 | 72.36 | 21.57 | Benign | Benign |
| | | | | | | | LN51 | 0.6 | Lateral | 0.05 | 0.05 | 0.05 | 4.57 | Benign | Benign |
| | | | | | | | LN52 | 1.2 | Lateral | 0.05 | 0.05 | 0.05 | 1.82 | Metastatic | Metastatic |
| Patient19 | No | Male | 26 | 25.5 | 1.2 | 0.01 | LN53 | 1.0 | Lateral | 0.05 | 0.05 | 0.05 | 0.26 | Benign | Benign |
| | | | | | | | LN54 | 1.1 | Lateral | 152.53 | 194.56 | 274.6 | 481 | Metastatic | Metastatic |
| Patient20 | No | Female | 32 | 24 | 0.4 | 0.4 | LN55 | 0.2 | Central | 162.74 | 241.07 | 299.87 | 481 | Metastatic | Benign |
| | | | | | | | LN56 | 0.2 | Central | 0.05 | 0.05 | 0.05 | 2.59 | Benign | Benign |
| Patient21 | No | Male | 52 | 23.5 | 0.4 | 71.7 | LN57 | 0.4 | Central | 6.12 | 8.37 | 21.85 | 0.34 | Benign | Benign |
| | | | | | | | LN58 | 0.7 | Central | 0.05 | 0.05 | 0.05 | 110.83 | Benign | Benign |
| Patient22 | No | Female | 23 | 19.4 | 0.7 | 331.69 | LN59 | 0.7 | Central | 0.05 | 0.05 | 0.05 | 1.57 | Benign | Benign |
| | | | | | | | LN60 | 0.9 | Central | 0.05 | 0.05 | 0.05 | 2.74 | Benign | Benign |
| | | | | | | | LN61 | 0.8 | Central | 6.77 | 48.95 | 95.1 | 75.32 | Metastatic | Metastatic |
| Patient23 | No | Male | 34 | 30.4 | 1.5 | 0.76 | LN62 | 0.5 | Central | 86.75 | 131.29 | 184.3 | 481 | Metastatic | Metastatic |
| | | | | | | | LN63 | 0.5 | Central | 29.66 | 53.44 | 85.42 | 481 | Metastatic | Metastatic |
| | | | | | | | LN64 | 0.6 | Lateral | 1.68 | 2.3 | 3.17 | 8.93 | Benign | Benign |
| Patient24 | No | Female | 52 | 26.7 | 1.9 | 0.14 | LN65 | 0.6 | Lateral | 0.05 | 0.05 | 0.05 | 0.15 | Benign | Benign |
| | | | | | | | LN66 | 0.6 | Lateral | 138.6 | 192.47 | 224.62 | 410.95 | Metastatic | Benign |
| Patient25 | No | Female | 46 | 23.1 | 1.1 | 35.68 | LN67 | 0.6 | Central | 33.13 | 66.58 | 87.52 | 72.92 | Metastatic | Benign |
| | | | | | | | LN68 | 0.4 | Central | 13.19 | 44.45 | 77.17 | 37.15 | Benign | Benign |
| Patient26 | No | Female | 31 | 21.5 | 1.4 | 1.12 | LN69 | 0.2 | Central | 3.63 | 4.75 | 8.64 | 75.45 | Benign | Benign |
| | | | | | | | LN70 | 0.5 | Central | 4.23 | 6.39 | 8.8 | 22.53 | Benign | Benign |
| Patient27 | No | Male | 44 | 24.5 | 1.5 | 75.44 | LN71 | 1.2 | Lateral | 0.05 | 0.05 | 0.05 | 2.77 | Benign | Benign |
| | | | | | | | LN72 | 1.0 | Lateral | 37.76 | 51.58 | 109.63 | 481 | Metastatic | Metastatic |

| | | | | | | | | | | | | | | | |
|---|---|---|---|---|---|---|---|---|---|---|---|---|---|---|---|
| Patient28 | No | Female | 55 | 29.5 | 0.8 | 46.38 | LN73 | 1.4 | Lateral | 75.07 | 127.43 | 169.28 | 481 | Metastatic | Metastatic |
| | | | | | | | LN74 | 0.6 | Central | 0.68 | 1.09 | 2.22 | 65.94 | Benign | Benign |
| | | | | | | | LN75 | 0.4 | Central | 3.34 | 5.08 | 6.33 | 31.66 | Benign | Benign |
| Patient29 | No | Male | 46 | 28.4 | 3.0 | 0.1 | LN76 | 1.0 | Lateral | 75.32 | 121.05 | 189.74 | 481 | Metastatic | Metastatic |
| | | | | | | | LN77 | 1.1 | Central | 1.05 | 1.86 | 4.3 | 27.96 | Benign | Benign |
| | | | | | | | LN78 | 0.4 | Lateral | 368.4 | 442.8 | 462.9 | 481 | Metastatic | Metastatic |
| Patient30 | Yes | Female | 25 | 18.8 | 3.0 | 4.5 | LN79 | 0.8 | Lateral | 25.33 | 54.89 | 87.4 | 481 | Metastatic | Metastatic |
| | | | | | | | LN80 | 0.8 | Lateral | 43.35 | 75.59 | 127.83 | 481 | Metastatic | Metastatic |
| | | | | | | | LN81 | 0.6 | Lateral | 34.38 | 67.78 | 129.69 | 481 | Metastatic | Metastatic |
| Patient31 | No | Female | 27 | 21.6 | 2.5 | 0.01 | LN82 | 1.1 | Lateral | 37.56 | 52.07 | 118.97 | 481 | Metastatic | Metastatic |
| | | | | | | | LN83 | 1.0 | Lateral | 41.4 | 60.47 | 93.22 | 481 | Metastatic | Metastatic |
| | | | | | | | LN84 | 0.5 | Lateral | 83.22 | 137.2 | 186.42 | 481 | Metastatic | Benign |
| | | | | | | | LN85 | 0.8 | Lateral | 86.5 | 150.2 | 220.61 | 481 | Metastatic | Metastatic |
| Patient32 | No | Female | 37 | 20.4 | 3.2 | 1.9 | LN86 | 0.8 | Lateral | 0.05 | 0.05 | 0.05 | 1.99 | Benign | Benign |
| | | | | | | | LN87 | 1.8 | Lateral | 0.05 | 0.05 | 0.05 | 5.97 | Benign | Benign |
| | | | | | | | LN88 | 0.9 | Lateral | 39.87 | 61.07 | 94.12 | 481 | Metastatic | Metastatic |
| | | | | | | | LN89 | 1.0 | Lateral | 0.05 | 0.05 | 0.05 | 1.45 | Benign | Benign |
| Patient33 | No | Female | 42 | 22.9 | 0.8 | 481 | LN90 | 0.8 | Central | 1.98 | 2.96 | 3.22 | 37.11 | Benign | Benign |
| | | | | | | | LN91 | 0.8 | Central | 23.9 | 36.46 | 67.12 | 15.63 | Benign | Benign |
| | | | | | | | LN92 | 0.8 | Central | 0.05 | 0.05 | 0.05 | 15.39 | Benign | Benign |
| | | | | | | | LN93 | 0.4 | Central | 0.05 | 0.05 | 0.05 | 12.39 | Benign | Benign |
| Patient34 | No | Female | 51 | 28.7 | 2.7 | 0.34 | LN94 | 1.2 | Lateral | 24.57 | 55.77 | 83.96 | 185.76 | Metastatic | Metastatic |
| | | | | | | | LN95 | 0.9 | Lateral | 97.47 | 202.95 | 224.61 | 481 | Metastatic | Metastatic |
| | | | | | | | LN96 | 1.5 | Lateral | 56.14 | 151.59 | 207.79 | 481 | Metastatic | Metastatic |
| | | | | | | | LN97 | 1.1 | Lateral | 0.05 | 6.38 | 13.88 | 49.75 | Benign | Metastatic |

| | | | | | | | | | | | | | | | |
|---|---|---|---|---|---|---|---|---|---|---|---|---|---|---|---|
| | | | | | | | LN98 | 0.6 | Lateral | 229.5 | 273.25 | 320.36 | 481 | Metastatic | Metastatic |
| Patient35 | No | Male | 43 | 24.8 | 2.0 | 0.05 | LN99 | 1.4 | Central | 15.52 | 20.54 | 28.06 | 481 | Metastatic | Metastatic |
| | | | | | | | LN100 | 0.6 | Lateral | 178.14 | 216.57 | 267.18 | 481 | Metastatic | Metastatic |
| | | | | | | | LN101 | 1.8 | Lateral | 326.49 | 358.82 | 429.45 | 481 | Metastatic | Metastatic |
| Patient36 | No | Female | 56 | 26.4 | 3.5 | 27.69 | LN102 | 0.5 | Lateral | 1.54 | 2.54 | 3.79 | 0.08 | Benign | Metastatic |
| | | | | | | | LN103 | 0.6 | Lateral | 0.05 | 0.05 | 0.05 | 0.04 | Benign | Metastatic |
| | | | | | | | LN104 | 1.8 | Central | 34.64 | 54.77 | 85.01 | 481 | Metastatic | Metastatic |
| Patient37 | No | Male | 34 | 26 | 1.8 | 0.03 | LN105 | 0.9 | Lateral | 96.17 | 223.56 | 308.74 | 481 | Metastatic | Metastatic |
| | | | | | | | LN106 | 1.1 | Lateral | 185.13 | 310.01 | 393.77 | 481 | Metastatic | Benign |
| | | | | | | | LN107 | 0.6 | Lateral | 4.83 | 7.34 | 12.8 | 108.79 | Benign | Benign |
| | | | | | | | LN108 | 0.8 | Lateral | 54.29 | 79.18 | 142.34 | 159.96 | Benign | Benign |
| Patient38 | Yes | Female | 55 | 25.4 | 2.1 | 0.08 | LN109 | 0.7 | Lateral | 25.5 | 75.43 | 136.12 | 159.15 | Metastatic | Metastatic |
| | | | | | | | LN110 | 0.7 | Lateral | 41.96 | 72.95 | 128.31 | 481 | Metastatic | Metastatic |
| | | | | | | | LN111 | 1.9 | Lateral | 41.98 | 81.86 | 133.75 | 157.7 | Metastatic | Metastatic |
| | | | | | | | LN112 | 0.7 | Lateral | 0.05 | 0.05 | 0.05 | 2.37 | Benign | Benign |
| Patient39 | No | Female | 33 | 19.1 | 2.9 | 3.58 | LN113 | 1.2 | Lateral | 0.05 | 0.05 | 0.05 | 0.64 | Benign | Benign |
| | | | | | | | LN114 | 0.7 | Lateral | 0.05 | 0.05 | 0.05 | 0.67 | Benign | Benign |
| | | | | | | | LN115 | 0.2 | Lateral | 0.05 | 0.05 | 0.05 | 1.74 | Benign | Benign |
| | | | | | | | LN116 | 1.1 | Lateral | 0.05 | 0.05 | 0.05 | 2.73 | Benign | Benign |
| Patient40 | Yes | Male | 26 | 28.9 | 0.7 | 481 | LN117 | 1.0 | Lateral | 96.65 | 190.02 | 249.85 | 481 | Metastatic | Metastatic |
| | | | | | | | LN118 | 1.2 | Central | 14.21 | 17.51 | 27.81 | 481 | Metastatic | Metastatic |
| | | | | | | | LN119 | 0.6 | Lateral | 33.07 | 194.56 | 270.08 | 481 | Metastatic | Metastatic |
| Patient41 | No | Male | 35 | 27 | 0.5 | 25.76 | LN120 | 0.5 | Central | 0.05 | 0.05 | 0.05 | 1.49 | Benign | Benign |
| | | | | | | | LN121 | 0.5 | Central | 15.58 | 27.63 | 48.54 | 10.15 | Benign | Benign |
| Patient42 | No | Female | 34 | 18.4 | 0.7 | 3.89 | LN122 | 0.6 | Central | 0.05 | 3.33 | 4.75 | 51.36 | Benign | Benign |

| | | | | | | | | | | | | | | | |
|---|---|---|---|---|---|---|---|---|---|---|---|---|---|---|---|
| Patient43 | No | Male | 24 | 20 | 0.4 | 0.01 | LN123 | 0.9 | Central | 4.01 | 5.54 | 6.46 | 52.97 | Benign | Benign |
| | | | | | | | LN124 | 0.5 | Central | 0.05 | 0.05 | 0.05 | 19.54 | Benign | Benign |
| | | | | | | | LN125 | 0.3 | Central | 0.05 | 3.81 | 5.66 | 41.52 | Benign | Benign |
| | | | | | | | LN126 | 0.3 | Central | 67.81 | 112.32 | 174.65 | 479 | Benign | Benign |
| Patient44 | No | Female | 51 | 28.7 | 1.5 | 0.01 | LN127 | 0.6 | Central | 0.05 | 6.39 | 34.18 | 190.27 | Metastatic | Metastatic |
| | | | | | | | LN128 | 0.6 | Central | 31.25 | 157.88 | 216.18 | 479 | Metastatic | Metastatic |
| Patient45 | No | Female | 51 | 25.4 | 1.1 | 481 | LN129 | 0.5 | Central | 46.98 | 92.63 | 153.34 | 479 | Metastatic | Metastatic |
| | | | | | | | LN130 | 0.5 | Central | 0.05 | 6.1 | 27.6 | 95.6 | Benign | Benign |
| | | | | | | | LN131 | 0.4 | Central | 4.05 | 7.37 | 11.22 | 201.41 | Benign | Benign |
| | | | | | | | LN132 | 0.4 | Central | 0.05 | 5.37 | 18.94 | 405.64 | Benign | Benign |
| Patient46 | No | Male | 46 | 25.6 | 0.4 | 0.01 | LN133 | 0.5 | Central | 40.77 | 68.63 | 98.37 | 100.9 | Benign | Benign |
| | | | | | | | LN134 | 0.4 | Central | 59.34 | 98.93 | 111.14 | 290.87 | Benign | Benign |
| Patient47 | No | Female | 49 | 23.2 | 0.8 | 0.1 | LN135 | 0.5 | Central | 1.86 | 5.81 | 9.72 | 42.15 | Benign | Benign |
| | | | | | | | LN136 | 0.5 | Central | 0.05 | 3.26 | 5.79 | 1.85 | Benign | Benign |
| | | | | | | | LN137 | 0.4 | Lateral | 0.05 | 0.05 | 0.05 | 0.83 | Benign | Benign |
| | | | | | | | LN138 | 0.7 | Lateral | 1.94 | 3.38 | 7.62 | 10.02 | Metastatic | Benign |
| | | | | | | | LN139 | 0.4 | Central | 92.23 | 104.63 | 119.01 | 479 | Benign | Benign |
| Patient48 | No | Male | 36 | 24.2 | 0.9 | 0.2 | LN140 | 0.3 | Central | 0.05 | 0.05 | 0.05 | 12.32 | Benign | Benign |
| | | | | | | | LN141 | 0.3 | Central | 0.05 | 2.68 | 4.31 | 8.91 | Benign | Benign |
| Patient49 | No | Female | 60 | 23.7 | 1.5 | 0.01 | LN142 | 0.5 | Central | 0.05 | 0.05 | 0.05 | 1.47 | Benign | Benign |
| | | | | | | | LN143 | 0.5 | Central | 0.05 | 5.69 | 9.18 | 54.37 | Benign | Benign |
| | | | | | | | LN144 | 0.5 | Central | 1.38 | 6.81 | 14.5 | 58.05 | Benign | Benign |
| Patient50 | No | Male | 49 | 24.5 | 0.5 | 0.01 | LN145 | 0.4 | Central | 0.05 | 1.94 | 3.29 | 14.62 | Benign | Benign |
| | | | | | | | LN146 | 0.4 | Central | 0.05 | 0.05 | 0.05 | 12.28 | Benign | Benign |
| Patient51 | No | Female | 38 | 19.1 | 0.8 | 67.17 | LN147 | 0.5 | Central | 28.09 | 58.36 | 86.72 | 479 | Metastatic | Metastatic |

| | | | | | | | | |
|---|---|---|---|---|---|---|---|---|
| LN148 | 0.3 | Central | 111.53 | 236.48 | 313.25 | 479 | Metastatic | Metastatic |
| LN149 | 0.5 | Central | 78.45 | 169.06 | 216.75 | 479 | Metastatic | Metastatic |
| LN150 | 0.4 | Central | 41.1 | 58.73 | 85.57 | 479 | Benign | Metastatic |
